# Blood utilization and quality indicators at a university hospital in the Eastern Province of Saudi Arabia

**DOI:** 10.1371/journal.pone.0267449

**Published:** 2022-04-22

**Authors:** Hwazen Shash, Rana Alabdulqader, Lama Alshehri, Norah Alkathery, Rozanna Al-Abdulrahman, Shatha Alahmed, Dalal Bubshait, Suzan AlKhater, Awatif Al-Nafie

**Affiliations:** 1 College of Medicine, Imam Abdulrahman Bin Faisal University, Dammam, Saudi Arabia; 2 Department of Pediatrics, King Fahad Hospital of the University, Al-Khobar, Saudi Arabia; 3 Department of Pathology, King Fahad Hospital of the University, Al-Khobar, Saudi Arabia; Qatar University, QATAR

## Abstract

**Background:**

Blood transfusion is a common, essential procedure when treating many different medical and surgical conditions. Efficient utilization of blood bank facilities by frequent auditing is crucial; however, few studies have examined blood utilization in Saudi Arabia. We aimed to review the blood ordering patterns and transfusion practices, and blood bank audit effectiveness at a single center in Saudi Arabia and compare our results with those of a similar study performed in the same center 20 years ago.

**Materials and methods:**

This study was a retrospective descriptive chart review of all healthy blood donors and recipients from January 1, 2016, to December 31, 2020. We evaluated the crossmatching-to-transfusion ratio (C/T) as an indicator of blood bank utilization and compared the findings with those of the previous study. We also evaluated changes in blood bank utilization during the coronavirus disease 2019 pandemic.

**Results:**

Findings from 27,414 donors (men, 94.9%; mean age, 32.2 + 9.6 years) showed a 71% increase in blood donations compared to that of 2000. The donations gradually increased over the years, peaking just before COVID-19 pandemic started in March 2020. For 3,836 patients, 13,324 units of blood were crossmatched (average, 3.47 crossmatch/patient), with 23% of the crossmatch requests from surgical departments. The average C/T ratio, transfusion index, and transfusion probability (T%) were 1.37, 2.55, and 73.2%, respectively. The C/T ratio decreased by 54% between 2000 and 2020. During the pandemic, crossmatching decreased by 26% between 2019 and 2020, but with comparable C/T ratio in 2019 (1.45) and 2020 (1.39).

**Conclusion:**

Our hospital blood bank utilization improved over the past 20 years, showing increased donations, reduced C/T ratio, and increased T%. This improvement emphasizes the importance of blood donation campaigns, blood bank auditing, restrictive transfusion guidelines, and physician education.

## Introduction

Transfusion of blood and blood products is a frequently performed practice during hospitalization. Demand for the control of blood utilization in transfusion medicine has increased, mainly due to the logistics of supply and demand, economics, and evidence-based medical practice with more restricted transfusion guidelines [1]. In Saudi Arabia, because blood products are collected from unpaid healthy volunteers, health care providers must rationalize its use [2]. Over-ordering of blood can lead to time and reagent wastage and an increase in blood bank cost and workload [3]. Constant monitoring of blood consumption and auditing of transfusion processes are required to identify major areas of concern in blood component utilization, allowing for the formulation of corrective actions [4].

Crossmatching of blood products results in a unit of blood being assigned to a particular patient; however, this is inaccessible for use for 48 to 72 hours. Delayed availability means there is a lower chance of it being used, leading to its expiration [5]. Several indicators are used to verify that blood and blood components are being used effectively. In 1975, Boral and Henry proposed the use of the crossmatch-to-transfusion (C/T) ratio, which is now a widely accepted indicator of blood bank utilization [6]. Other markers include transfusion probability percentage (T%) and transfusion index (TI). TI refers to the average number of units used per patient, which indicates the suitability of crossmatching the corresponding number of units [5]. In 1975, Friedman et al. introduced the maximum surgical blood order schedule (MSBOS) to reduce excessive crossmatching of units of blood products preoperatively for patients undergoing elective surgery [5]. The MSBOS is intended to link the number of units of blood crossmatched for a certain surgery with the number of units actually transfused to the patient more accurately [5]. It is modified by individual hospitals according to their policies and experience.

There are few studies from Saudi Arabia regarding blood bank utilization, and most include small sample sizes and short follow-up periods [2, 7–10]. A study on blood bank utilization conducted in our center between 1996 and 2000 showed an average C/T ratio of 2.96, which did not meet the international standards of less than 2, and over-ordering of blood products and cancellation of over 60% of transfusions [7, 11]. Before 1999, the standard of care involved “liberal” indications for blood transfusion; where the target hemoglobin was more than 10 gm/dL. However, this changed after the randomized controlled trial published in 1999 by Hébert et al. reported a “restrictive” strategy was just as effective provided it is correlated to the clinical presentation [12]. The initial study in our institution was done prior to the change in the standard of care. During this time, our transfusion committees established policies and procedures that were regularly updated according to the Association for the Advancement of Blood and Biotherapies (AABB) guidelines. Clinical departments were educated periodically to ensure implementation of the updated guidelines. The hospital obtained College of American Pathologists accreditation in 2012 and Joint Commission International accreditation in 2015. The international accreditations ensured that the hospital maintains the highest standards of care. Therefore, in the present study, we reviewed the utilization of blood products after an extensive audit and review of the blood bank’s policies and procedures. The transfusion policy currently followed in our hospital is based on the most recent AABB guidelines updates in 2016 [1].

This study aimed to review blood ordering patterns and transfusion practices in our hospital 20 years after the initial study and to evaluate the effectiveness of our blood bank auditing. We also evaluated the overall C/T ratio, T%, and TI in the whole hospital and for each specialty. We also evaluated the effect of the coronavirus disease 2019 (COVID-19) pandemic on our blood bank. We hoped that our findings will facilitate the implementation of improvements at our hospital.

## Materials and methods

### Study design

This study was a retrospective cohort chart review of all blood bank records of donors and recipients between January 1, 2016, and December 31, 2020, at the King Fahad Hospital of the University, Al-Khobar, Saudi Arabia. The Institutional Review Board approved this study (IRB-UGS-2020-01-293). Given the retrospective nature of the study, the requirement for informed consent was waived.

### Data collection

The data of the donors and recipients were obtained from paper records and electronic medical records, respectively. The data collected on the donors included age, sex, nationality, and the type of donation. The data collected from the tests performed on the blood units included ABO blood type, RhD, and antibody screening results. Infectious agent screening included hepatitis B surface antigen (HBsAg), hepatitis B core antibody (HBcAb), and hepatitis C virus antibody (HCV Ab). In cases where the HBcAb screening was positive, a test for hepatitis B surface antibody (HBsAb) was performed. The blood units were also screened for human immunodeficiency virus (HIV), syphilis [rapid plasma regain (RPR)], human T-cell lymphotropic virus antibody (HTLV1/2 Ab), and malaria antigen. Nucleic acid testing (NAT) and blood cultures were also performed. The reasons for discarding blood post-donation (infectious and non-infectious) were also noted. Donors who could not donate due to difficult venous access and platelet apheresis donors were excluded from the study.

Data collected on the recipients included age, sex, nationality, and the service department that ordered the crossmatch. The number of units requested and consumed, ABO/Rh grouping of both the transfused blood product and patient, the type of blood product requested [packed red blood cells (PRBC), fresh frozen plasma (FFP), platelets], and results of the direct antiglobulin test and antibody screen were also evaluated. We excluded patients who received cryoprecipitates and whole blood, as they were infrequently requested.

### Laboratory investigations

The ABO blood type, RhD, antibody screening, and crossmatch were performed using the DiaClon gel testing method (Bio-Rad Laboratories, Cressier, Switzerland). Serology testing for HBsAg, HBcAb, HBsAb, HCV Ab, HIV, and HTLV1/2 Ab was performed using the Alinity I Series (Abbott, Illinois, USA). RPR was performed using INNO-LIA Western blot (Innogenetics NV, Ghent, Belgium). Malaria antigen testing was performed using Malaria P.f/Pan Ag (Cypress Diagnostics, Hulshout, Belgium). NAT test was performed using Procleix Panther (Ilex, Gauteng, South Africa). All tests were performed according to the respective manufacturer instructions.

### Statistical analysis

Data from clinical records were analyzed using SPSS version 24 (IBM Corp., Armonk, NY, USA). Categorical data are presented as frequency and percentage, and continuous data as mean ± standard deviation (SD) or median with 25^th^–75^th^ [interquartile range (IQR)], as appropriate according to the data distribution. The C/T, T%, TI, and MSBOS (for surgical specialties) were calculated as follows:

- C/T = number of units crossmatched/number of units transfused
- TI = number of units transfused/number of patients crossmatched
- T% = number of patients transfused × 100/number of patients crossmatched
- MSBOS = 1.5 x TI

A C/T ratio ≤ 2 was considered effective blood usage. A value ≥ 0.5 was considered an effective TI, and a value ≥ 30% was regarded as normal T% [3].

## Results

### Donors

**Fig 1:**
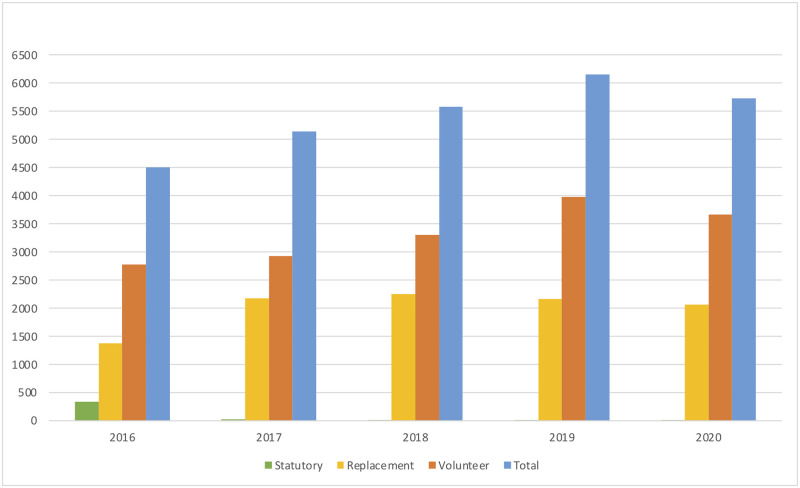
The number of blood donations per year from 2016–2020 by purpose of donation; statutory (green), replacement (yellow), volunteer (orange), and total (blue).

From January 1, 2016, to December 31, 2020, 27,904 donors visited the blood bank for donations. After excluding 490 donors due to difficult venous access, 27,414 donors were included in this study, of which 94.9% were men. The most common age group that donated was between 25–40 years (54.8%), followed by 18–24 years (25.6%). Saudis constituted 77% of the donors, and they were more likely to be volunteers compared to non-Saudis (63.1% and 56.8%, respectively). The main source of blood was from volunteers (61.5%), followed by replacement donors (37.1%) and statutory donors (1.4%). The frequency of donations increased during the study years, with a peak in 2019 (Fig 1). Statutory donations decreased from 337 donors in 2016 to only six donors in 2020.

In total, 3,183 units (11.6%) were Rh-negative. The most common blood type donated was O positive (45.2%), followed by A positive (22.83%) (Table 1). Of all the donors, 1,737 (6.3%) tested positive in the infectious agent screening. The most common infectious agent detected was HBcAb (4.9%) (Table 2).

**Table 1:** ABO/Rh blood donation types.

Blood type	Number (%)
A positive	6,258 (22.83)
A negative	672 (2.45)
B positive	4,505 (16.43)
B negative	496 (1.81)
O positive	12,396 (45.21)
O negative	1,899 (6.93)
AB positive	1,072 (3.9)
AB negative	116 (0.4)
Total	27,414 (100.0)

**Table 2:** Infectious screening markers for donors.

Screening test	Number	Positive units (% of total) (n = 27,414)	Infectious discards (%) (Total units discarded = 1,737)
HBsAg	65	0.2	3.8
HBcAb	1,341	4.9	77.2
HCV	100	0.4	5.6
HIV	21	0.10	1.2
RPR	106	0.4	6.1
HTLV 1/2	37	0.1	2.1
Malaria Ag	3	0.0	0.2
NAT	64	0.2	3.7

HbsAg: Hepatitis B surface antigen; HbcAb: Hepatitis B core antibody; HCV: Hepatitis C virus; HIV: Human immunodeficiency virus; RPR: rapid plasma regain; HTLV1/2: Human T-cell lymphotropic virus; Ag: antigen; NAT: nucleic acid testing.

**Fig 2:**
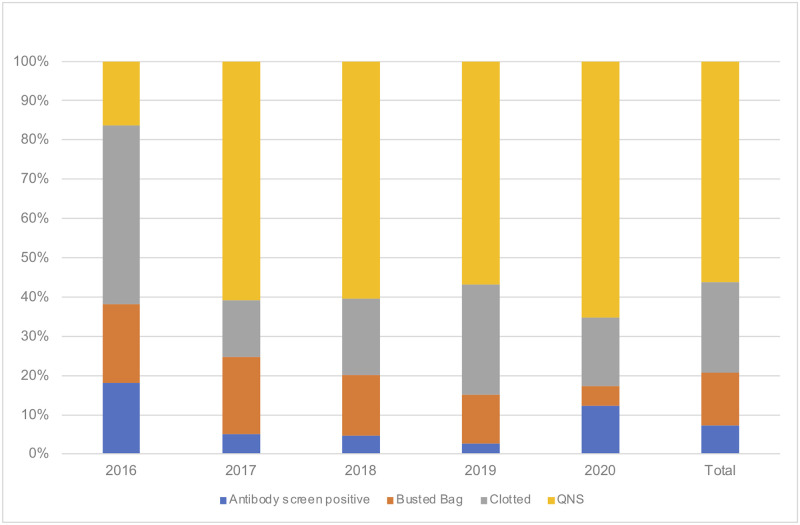
Non-infectious causes of discarding blood per year from 2016–2020 and total cohort. The percentage of antibody screen (blue), busted bag (orange), clotted units (grey), and quantity not sufficient (yellow). QNS: quantity not sufficient.

Of the collected units, 2,325 (8.5%) were discarded due to positivity in the infectious agent screen (74.7%), non-infectious causes (23.9%), and unknown causes (1.7%). Blood units that tested positive in the screening were discarded, regardless of the results of a confirmatory test. Insufficient quantity of blood collected was the most common (56%) non-infectious cause for discarding blood (Fig 2). The number of bags discarded due to insufficient quantity increased from 16% in 2016 to 65% in 2020. The frequency of discards decreased due to clotted units (from 45.6% in 2016 to 14.4% in 2017) and due to damaged bags (from 20% in 2016 to 5% in 2020).

Every donation produces one unit PRBC, while the FFP and platelets are supplied according to the demand of the hospital. The total number of units produced by the blood bank was 25,089 units of packed red blood cells, 9,234 units of fresh frozen plasma, and 13,902 units of platelets during the study years.

### Recipients

For the 3,836 patients anticipated to require a transfusion, a crossmatch was ordered 13,324 times (average, 3.47 crossmatch/patient). Each crossmatch request was accompanied by the number of units needed, which was in a median of 2 units per request (IQR 1–2). The mean age of the recipients was 31.1 + 23.1 years, and 64.6% of them were women (male:female ratio, 0.55:1). Most recipients (83.7%) were Saudis, and the most frequent (47.6%) blood type requested was O positive. The most frequently ordered blood components were packed PRBCs (77.2%), followed by FFP (12.9%) and platelets (10%). Table 3 summarizes the number of blood components requested and consumed and the C/T ratio for the different blood products for the whole cohort. The average C/T ratio, TI, and T% was 1.37, 2.55, and 73.2%, respectively (Table 3). Most of the requests for crossmatching were from surgical departments (23%). The lowest and highest C/T ratios were related to requests from the Departments of Medicine and Surgery, respectively (Table 4). The average MSBOS from surgery was 4 (Table 5).

**Table 3:** Crossmatch transfusion ratio according to blood product.

Blood Component Requested		Number of units requested [IQR/SD]	Number of units transfused	Transfusion probability	C/T ratio
PRBC	N	10,281	7,640	7,640 (74.3%)	1.35
	Median	2 [1–2]	1 [0–2]		
	Mean	1.75 [0.867]	1.13 [0.879]		
FFP	N	1,713	1,146	1,146 (66.9%)	1.49
	Median	2 [1–4]	1 [0–2]		
	Mean	2.94 [3.34]	1.80 [3]		
Platelets	N	1,330	962	962 (72.3%)	1.38
	Median	2 [1–4]	1 [0–3]		
	Mean	3.1 [2.32]	1.81 [1.86]		
Total	N	13,324	9,748	9,748 (73.2%)	1.37
	Median	2 [I1–2]	1 [0–2]		
	Mean	2 [1.68]	1.28 [1.48]		

Data are shown as mean ± standard deviation (SD) or median with 25^th^–75^th^ [interquartile range (IQR)], C/T: Crossmatch-to-transfusion ratio; PRBC: Packed red blood cells; FFP: Fresh frozen plasma.

**Table 4:** Quality indicators for blood bank utilization by specialty.

Specialty	Number of requests (% of total)	C/T Ratio	Transfusion probability (%)	Transfusion index (TI)
Medicine	2642 (19.8)	1.18	84.9	5.9
Surgery	3060 (23.0)	1.72	58.1	2.7
Pediatrics	2540 (19.1)	1.23	81.3	5.24
NICU	1992 (15.0)	1.34	75	4.7
Ob/Gyn	2061 (15.5)	1.52	65.6	1.5
ED	633 (4.8)	1.24	80.9	2.92
ICU	358 (2.7)	1.27	78.5	3.7
Others	38 (0.3)	1.58	63.2	1.58
Total	13,324 (100)	1.37	73.2	2.55

NICU: Neonatal intensive care unit; Ob/Gyn: Obstetrics and gynecology; ED: Emergency department; ICU: Intensive care unit.

**Table 5:** Quality indicators for blood utilization for transfusion in surgical subspecialties.

Specialty	Number of requests (%)	C/T Ratio	Transfusion Probability (%)	Transfusion Index (TI)	MSBOS
General	1701 (55.6)	1.72	58.3	1.71	2.57
Cardiothoracic	125 (4.1)	1.54	64.8	2	3
Neurosurgery	434 (14.2)	1.83	54.6	1.83	2.75
ENT	78 (2.5)	1.9	52.6	1	1.5
Plastic	63 (2.1)	1.58	63.5	2.35	2.53
Orthopedic	369 (12.1)	1.74	57.5	1.33	2
Urology	84 (2.7)	1.9	52.4	1.1	1.65
Pediatric	199 (6.5)	1.55	64.3	1.71	2.57
Ophthalmology	7 (0.2)	1.4	71.4	1.25	1.88
Total	3060	1.72	58.1	2.7	4

ENT: Ear, nose, throat.

### Impact of the COVID-19 pandemic

The number of donations increased by an average of 10% (SD +2%) per year from 2016–2019. However, the donations decreased by 7% between 2019 and 2020. Despite this decrease, we found that the number of donations were similar in 2018 (before pandemic) and 2020 (during the pandemic). We studied the monthly changes in 2020 considering the frequent pandemic-related changes in that year.

**Fig 3:**
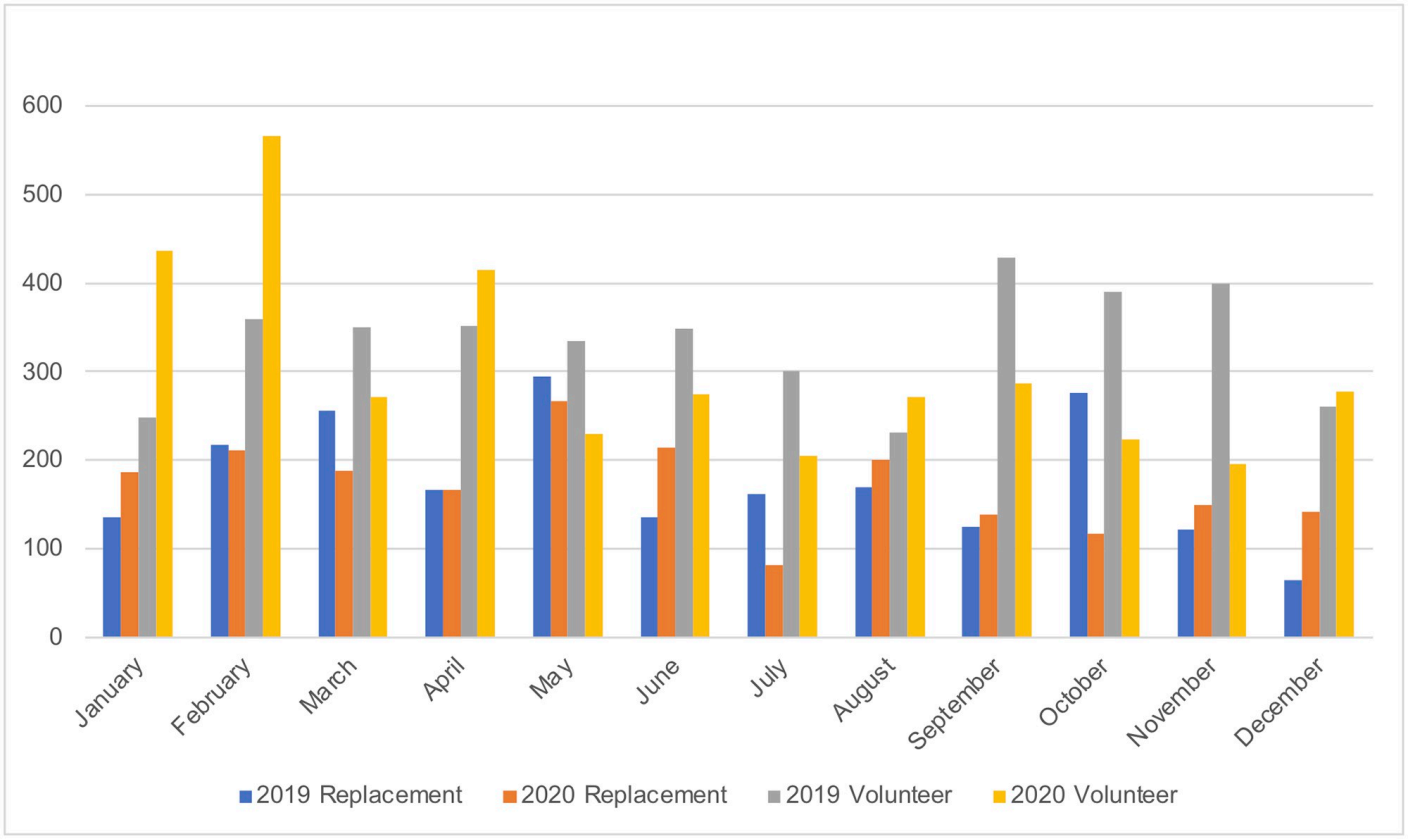
The frequency and purpose of blood donation by month in 2019 and 2020. The replacement for 2019 (blue), 2020 (orange) and volunteer in 2019 (grey), 2020 (yellow).

The number of volunteer donations decreased in March 2020, however, they increased again in April 2020 despite the 24-hour curfew in the city. Volunteer donations decreased from May through July 2020 and again from September through November 2020 compared to the same time period in 2019. Replacement donations decreased in March, July, and October 2020 compared to the same time period in 2019. The only month when replacement donations were greater than that of the volunteers was May 2020 (Fig 3).

**Fig 4:**
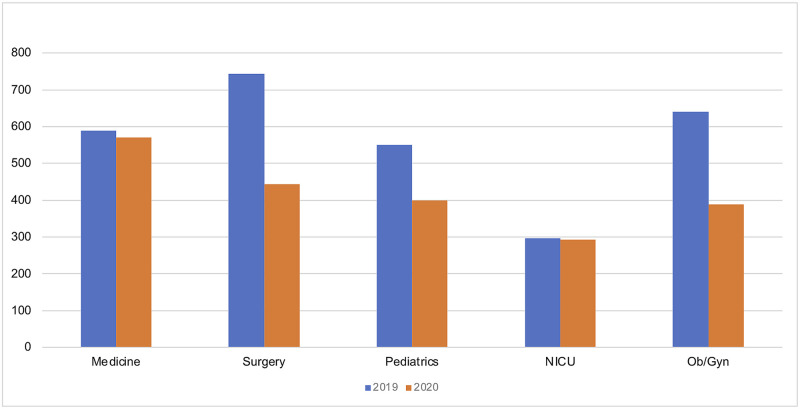
Frequency of crossmatch requests by department in 2019 (blue) and 2020 (orange). NICU: Neonatal intensive care unit; Ob/Gyn: obstetrics and gynecology.

Crossmatch requests decreased from 3,091 in 2019 to 2,270 in 2020 (26.6% decrease), while C/T ratios were comparable (1.45 in 2019 and 1.39 in 2020). The requests for blood transfusions decreased in all departments. The Department of Medicine and the neonatal intensive care had the least decrease in number of requests (3% and 1.4% respectively). Decreases of 40% and 39% were seen in the Department of Surgery and the Department of Obstetrics and Gynecology, respectively in 2020 (Fig 4).

## Discussion

Our study showed improvements in blood utilization services with a decrease in the average C/T ratio from 2.96 to 1.37 (a 54% decrease) between the two time periods [7]. The number of crossmatches decreased significantly from 46,088 (1996–2000) to 13,324 (2016–2020) (71.1% decrease) [7]. This decrease is attributed to physician education, more stringent indications for blood transfusion, and blood bank auditing.

People who donate blood freely out of compassion and are mindful of their health are the safest donors [13]. There has been an increase in voluntary blood donation worldwide, including in Saudi Arabia [14]. The frequency of blood donations has increased over the past 20 years. While the total number of blood donations has increased from 17,442 to 27,414 (36%), voluntary donations have increased from 18.9% to 61.5% [7]. This is likely due to the increased awareness, social media impact, and blood donation campaigns [15, 16]. In Saudi Arabia, blood bank opening hours have been extended to allow voluntary donors to be able to donate after work. There are also donation trucks in major areas of the cities, and campaigns where donation can occur in shopping malls. In addition, the Ministry of Health launched “Wateen,” Saudi Arabia’s official blood donation application, in March 2019 [17]. Wateen is the Arabic word for the aorta, which is the major artery in the human body. The application was created to facilitate the donation process, and it can be used by blood donors, recipients and their families, and blood bank representatives [17]. This application allows donors to locate blood banks in their area, follow donation records across different hospitals, and request a blood donation from the Wateen community [18]. Expatriates constitute about one-third of the population in Saudi Arabia and 23% of donors [19]. A study from a military hospital in the region found that most donations were for replacement and were donated by Saudis [13]. Unlike some hospitals that only cover a particular population, ours is a government university hospital covering the entire population and, therefore, it contains a diverse pool of donors and recipients.

Interestingly, men donated more often than women. While several studies showed a higher donation rate in men, others had the reverse findings, depending on the reporting country [20]. However, in Saudi Arabia, donations from women are low, which may be due to concerns with complications of donation, anemia, and difficult veins [21]. The donation campaigns may need to promote additional education to the female population targeting their concerns. The eastern region of Saudi Arabia has a high incidence of alpha thalassemia, making the baseline hemoglobin level low, which may cause deferral of donations [22].

Before donating blood products, a blood donor must meet several criteria [23]. These include physical standards like age, weight, and vital signs, as well as strict requirements around sexual, medical, and travel history. Any contraindications that surface during the interview and physical examination may result in temporary or permanent removal from the blood donation system. Our hospital complies with the international recommendations for the screening of donors [23]. Any donation with a positive screening test is discarded, regardless of the outcomes of confirmatory testing. Donors are informed of a positive screening test result only if the confirmatory tests are positive. However, those with unconfirmed positive screening tests are not informed, so they may donate again. The donation files are paper-based, and no specific identification number is given to the donor for repeated donations. Hence, the same donor could donate blood more than once, leading to an overestimation of the positive screening test results. For the same reason, it was difficult to determine the number of donations made by the same individual at our hospital. In the future, the “Wateen” application may provide a unified donor registry, including access to records of donors with rare blood types and deferred donors with reasons known to the hospitals.

The most common infectious cause for discarding blood was HBcAb positivity. The prevalence of hepatitis B virus (HBV) infections (indicated by the HBsAg results) in different age groups in Saudi Arabia has decreased since introduction of the universal vaccination program in 1989, when the overall prevalence was 6.7% [24]. Despite the high rate of HBcAb positivity in our cohort, the prevalence of HBsAg positivity was only 0.2%, indicating a marked reduction in the incidence of HBV infection. In a 2017 study conducted in primary health centers, hepatitis HCV seroprevalence was estimated to be 0.38%, with a higher prevalence attributed to those aged > 37 years, which is consistent with our findings of HCV positivity (0.4%) [25].

A wide range of C/T ratios have been reported, ranging from 1.0 to 6.0 [2]. Using the C/T ratio as a criterion, studies from several nations have reported excessive blood utilization (C/T ratio > 2) such as Ethiopia (2.3), Zambia (2.8), Tanzania (3.7), Egypt (3.9), and Malaysia (5) [2]. The C/T ratio can be evaluated for an institution as a whole or for each department. A C/T ratio of ≤ 2.0 is considered appropriate for surgical patients and around 1 for non-surgical patients when evaluating blood-ordering practices [26]. The C/T ratios in our hospital were consistent with the international standards for blood centers. While the overall C/T ratio for the hospital was 1.37, it was 1.18 and 1.72 for the Departments of Medicine and Surgery, respectively. The T% was 84.9% and 58.1% for the Departments of Medicine and Surgery, respectively, indicative of an optimal T%. A previous publication from our center reported an average C/T ratio of 2.96 (2.73–3.17), with no analysis of different specialties [7]. The marked decrease in the C/T ratio over the past 20 years in our hospital, associated with increased donation frequency, highlights the importance of auditing, communication with the blood bank, stringent indications for transfusions, and physician education.

The MSBOS can optimize the C/T ratio by reducing the excessive crossmatching of blood for patients undergoing elective surgery. The shorter time a unit of blood spends in a reserved (crossmatched) phase, the more readily available it is for transfusion [5]. However, some of the concerns regarding MSBOS are that the recommendations are frequently outdated, based on opinion, do not cover recently developed surgical techniques, and are not based on institution-specific blood usage statistics [27]. In the absence of an explicit MSBOS, blood transfusion orders usually rely on the physician’s experience and a subjective prediction of blood loss rather than on audit-based estimations of demand for a specific operation [28]. It was found that while certain centers always utilized blood for certain procedures, others did not for the same procedures in patients with identical clinical symptoms [29]. Therefore, it appears that blood transfusion is based on a physician’s request and not the patient’s need [29]. An optimal MSBOS can improve blood utilization in any institution by standardizing the use of blood in surgical procedures and reducing unnecessary requests.

For decades, there has been much debate on whether donor-recipient compatibility should be assessed by crossmatching (including an antiglobulin test of recipient’s serum vs. donor’s red cells) or a “type and screen” procedure [30]. Many hospitals have improved their C/T ratios by implementing an electronic crossmatch (EXM) with different guidelines [31]. While all centers follow the same general recommendations, the timing is different. To be eligible for EXM, the patient must have at least two separate blood samples where ABO/Rh tests were performed, a negative antibody screen, and no previously positive antibody screening test [31–33]. Patients with confirmed or suspected red cell alloantibodies, newborns, and recipients of ABO-incompatible hematopoietic stem cells are excluded from EXM [31–33].

The EXM was implemented in Sweden in the 1980s, and it has gained global popularity since then [30]. With EXM, C/T ratios of around 1.2 can be achieved [31]. It was found to reduce the workload in the lab, the number of blood components transferred to the ward, and the number of unused units [30, 31]. Compared to standard serological crossmatching, EXM offers several advantages, including a short processing time, low cost, and ease of use [32]. Surgeons believe that the advantages include promptness and a practically limitless supply of blood. When blood can be selected rapidly without delay, the necessity for reserving that blood diminishes, significantly lowering the amount of reserved blood [33]. EXM machines have evolved into "vending machines" for blood, located in operating room suites and electronically linked to the blood bank by a software interface [27]. Frank et al reported a 38% reduction in preoperative blood orders with the combined use of EXM and MSBOS [27]. Additionally, EXM can improve the safe transfer of blood products between hospitals.

The COVID-19 global pandemic caused a worldwide shortage of blood products, particularly when convalescent plasma was considered for managing severe cases [34]. Donor attendance dropped in several nations, including Washington, USA (10–30%), and Canadian Blood Services (30%) [35]. A study from a different region in Saudi Arabia reported setbacks with blood bank shortage issues [36]. However, in the early stages of the pandemic, elective surgeries and medical admissions were restricted in many centers. As a result, the decrease in demand for blood compensated for the decrease in blood donation. In our center, during the early phase of the pandemic, there was a large influx of voluntary donors since there were no mobile blood banks in operation. The large influx of donors in April 2020 may have been due to concerns about the severity of an unknown disease in the early phase of the pandemic, and the increased need for intensive care management of patients. The community united against the disease and donations increased to support others. Some other hospitals in the region sent mobile blood banks to a donor’s house to collect blood. Surprisingly, after the lockdown was lifted, voluntary donations decreased, perhaps due to a fear of exposure to other community members. These observations underscore the compassion within the community for others and the benefits of social media for encouraging blood donation [15, 16]. In our hospital, the crossmatch requests decreased by 26.6% between the year 2019 and the year 2020. The Department of Medicine had only a 3% decrease in crossmatches, likely due to the increased number of patients admitted to the medical intensive care with COVID-19 infection. It was reported between 3.3–13.4% of the patients with COVID-19 infection require blood transfusions. [35, 37, 38]. Discontinuing elective surgeries and elective admissions in our institution reduced the requirement for blood transfusions, as indicated by a decrease in crossmatches requested by the Department of Surgery by 40% between the year 2019 and the year 2020. These changes highlight the adequacy of our hospital management during the pandemic in making the blood bank more efficient with minimal deficiencies.

Our study has some limitations. First, the paper-based records for the donors had a higher incidence of missing data compared to the electronic data collection for the recipients. Second, every donation made was recorded on a different form, which made it difficult to identify repeated donors. Third, the crossmatch request form did not include a reason for transfusion, making it challenging to determine the exact indication of ordering the blood product. Lastly, there was no link between the donor and recipient files, so we could not trace the blood donation to the recipient. The use of a computerized system in the future would allow matching of blood product utilization data with clinical data and enable easy retrieval and management of sizable quantities of data.

## Conclusions

Our hospital’s blood bank utilization has improved over the past 20 years, as indicated by the reduction in C/T ratio and increase in T%. This improvement underscores the importance of blood bank auditing and physician education, and encourages hospitals to achieve comparable outcomes. We recommend that our hospital develop its own MSBOS to improve the efficiency of blood ordering and utilization. Additionally, we advocate laying the groundwork for a unified Saudi National Blood Transfusion Service to oversee future improvements in blood transfusion by establishing a central blood bank for all the hospitals (governmental, university, private) to improve services in the region. This may be initiated by utilizing a central software integrated with the Wateen application for donor tracing and introduction of EXM. We recommend conducting future prospective studies to observe the utilization of EXM in Saudi Arabian blood banks.

## Abbreviations

C/T: crossmatch-to-transfusion (C/T) ratio
COVID-19: coronavirus disease 2019
EXM: electronic crossmatch
FFP: fresh frozen plasma
HBcAb: hepatitis B core antibody
HBsAb: hepatitis B surface antibody
HBsAg: hepatitis B surface antigen
HCV Ab: hepatitis C virus antibody
HIV: human immunodeficiency virus
HTLV1/2 Ab: human T-cell lymphotropic virus
MSBOS: maximum surgical blood order schedule
NAT: Nucleic acid testing
PRBC: packed red blood cells
RPR: rapid plasma regain
T%: transfusion probability percentage
TI: transfusion index
